# CUCT-Net: End-to-End Signal-to-Image Learning for Quantized Speed-of-Sound Estimation and Tissue Segmentation in Ultrasound Computed Tomography

**DOI:** 10.3390/s26092801

**Published:** 2026-04-30

**Authors:** Qinhan Gao, Mohamed Khaled Almekkawy

**Affiliations:** School of Electrical Engineering and Computer Science, Penn State University, University Park, PA 16802, USA; qxg7@psu.edu

**Keywords:** deep learning, signal processing, ultrasound computed tomography, convolutional neural network

## Abstract

Objective: Traditional Full Waveform Inversion (FWI) methods for Ultrasound Computed Tomography (UCT) are computationally expensive and can be sensitive to strong acoustic contrasts. In this work, we propose the Multi-Channel Transducer Network (CUCT-Net), a deep learning framework that directly maps received ultrasound signals to image-space outputs for quantized speed-of-sound (SoS) estimation and for direct tissue-level segmentation over both low- and high-contrast regions, enabling end-to-end recovery of both contrast-driven and anatomically meaningful structures from raw measurements. Method: CUCT-Net uses a multi-input encoder–decoder architecture that maps raw multi-static UCT measurements to quantized SoS (or tissue-class) maps without requiring an initial guess or iterative optimization. Parallel per-transducer encoders extract view-specific features that are fused and refined by a decoder, with Shift Units (SU) used to enhance fine-scale feature modeling under sparse sensing. Experiments are performed on k-Wave simulations using (i) Shepp–Logan-inspired disc phantoms with Original/Distorted/Mixed variants and (ii) DBB-derived anatomical brain phantoms, under clean and noisy measurement conditions. Results: The proposed network achieves accurate quantized SoS estimation and direct tissue-level segmentation across synthetic and anatomically derived phantom experiments. Strong robustness to noise is demonstrated through transfer learning. Compared with FWI, CUCT-Net significantly reduces computational cost while maintaining stable performance under reduced-sensor conditions for quantized SoS estimation and complex tissue heterogeneity for segmentation. Conclusions: CUCT-Net formulates UCT as a direct signal-to-image learning problem that supports both quantized SoS estimation and tissue-level segmentation. By learning an end-to-end mapping from raw ultrasound measurements to quantized SoS or tissue representations, the proposed framework bypasses iterative inversion and achieves efficient and robust performance under reduced-sensor and strong-contrast conditions. The multi-input architecture enables effective integration of information from multiple transducers, demonstrating the feasibility and potential of data-driven end-to-end quantized SoS estimation and tissue segmentation for UCT.

## 1. Introduction

Ultrasound computed tomography (UCT) [1,2,3,4,5] has emerged as an advanced diagnostic modality that offers the potential for high-resolution images of soft and hard tissue structures. Traditional ultrasound imaging, which is invaluable owing to its real-time imaging capabilities and the absence of ionizing radiation, is sometimes limited by its reliance on operator skill and the acoustic impedance of the tissues. B-mode ultrasound may face challenges in distinguishing various tissue types or in identifying minor differences in tissue density [6]. In contrast, UCT enhances tissue characterization and contrast by leveraging its ability to measure acoustic properties such as the speed-of-sound (SoS) and attenuation. Among these, SoS plays a particularly crucial role, as it provides a direct link to the mechanical and structural properties of tissues, enabling more accurate identification of tissue types and anomalies [7]. Unlike traditional ultrasound, which primarily relies on backscattered signals to infer structural information, SoS-based imaging in UCT offers a quantitative measure that can distinguish subtle differences in tissue composition. This makes it especially effective for detecting anomalies, such as tumors, cysts, or abnormal growths, where variations in SoS values reveal pathological changes that may go undetected in conventional ultrasound imaging [8]. By integrating this level of precision, UCT facilitates more detailed and reliable tissue characterization, paving the way for improved diagnostic accuracy and clinical outcomes.

Generating UCT images from recorded transducer signals is a complex process that involves heavy computations. UCT image reconstruction typically relies on Iterative Reconstruction (IR) algorithms, which progressively refine images to improve their accuracy. Unlike direct reconstruction methods that attempt to solve the imaging problem in a single step (such as the inverse Radon transform in X-ray CT), IR approaches this problem by iterating between the measurement and image domains and making incremental improvements to the image with each cycle. Among IR methods, Full-Waveform Inversion (FWI) [9,10,11] is a refined approach that incorporates wave diffraction and reflection into the image-reconstruction process. By considering these complex wave phenomena, FWI enhances the spatial resolution of UCT images, providing detailed maps of tissue properties that simpler methods may overlook. This makes it a powerful tool for capturing fine structural details and accurately reconstructing tissue distribution.

However, FWI poses several challenges owing to its complex optimization process. The method seeks to determine the SoS distribution that best aligns with the recorded signals; however, this optimization is inherently large-scale, ill-posed, and nonlinear. Additionally, high contrast in SoS values between different tissue types can exacerbate these issues by increasing the sensitivity to local minima and making convergence more difficult, particularly in reduced-sensor or limited-view configurations.

Owing to the heavy computation time required by traditional FWI methods, deep learning has emerged as a promising alternative for UCT. Deep learning has revolutionized medical imaging by offering advanced techniques for tasks such as image reconstruction, diagnosis, and treatment planning [12,13,14,15]. Its ability to identify patterns and enhance image quality [16] has made it particularly effective in addressing challenges within UCT.

In the field of UCT, deep learning methods have demonstrated significant potential for improving reconstruction quality while reducing computational demands. For example, the method proposed in [17,18] aims to achieve reconstruction quality of FWI while maintaining computational efficiency. This approach uses low-resolution SoS images from ray-based travel time tomography and reflectivity data from reflection tomography as the inputs. SoS images provide information about tissue properties, whereas reflectivity data provide boundary details. These dual inputs were processed using a U-Net-based image reconstruction method trained to map them to high-resolution SoS images. This model captures intermediate mapping knowledge and enables accurate and efficient reconstruction.

Beyond this example, other deep-learning approaches in UCT have targeted specific challenges. In [19], the authors addressed the generalization of neural networks from simulated to real data by integrating Fourier transforms into a network. This integration improves the robustness to noise and enhances the generalizability of real-world measurements. Similarly, the method proposed in [20], called SRSS-Net, focuses on reconstructing SoS images from sparse data. By leveraging a convolutional neural network (CNN), SRSS-Net effectively suppresses artifacts, preserves structural details, and surpasses traditional methods in terms of accuracy and computational speed.

The study in [21] introduced a network architecture for large receptive fields by incorporating multiple downscaling and upscaling convolutional units. This architecture, trained on ImageNet and tested on an Optical and Acoustic Breast Phantom Database, demonstrated superior efficiency and robustness, delivering comparable or better quality than traditional methods. Meanwhile, ref. [22] explored limited-angle UCT for prostate cancer diagnosis, utilizing an autoencoder-like network to encode Time-of-Flight (ToF) data into lower-dimensional representations and decode them into SoS images. This method achieved high accuracy, demonstrating its potential for specialized applications.

Yan et al. [23] proposed an untrained-network full-waveform inversion (UNN-FWI) framework for breast UCT. Their method optimizes an encoder–decoder generator—initialized with random weights—for each slice so that its output, when passed through the acoustic forward model, minimizes the waveform data misfit. This slice-specific optimization yields impressive quantitative accuracy within the typical soft-tissue range of the breast and provides a physics-informed alternative to fully supervised learning.

Another significant contribution is presented in DeepUCT [24], where the central objective is to directly learn mapping from the recorded time-series sensor data to a spatial image representing acoustical properties. To achieve this, DeepUCT comprises two cascaded CNNs with an encoder–decoder architecture. This approach emphasizes the direct utilization of raw sensor data, eliminating the need for extensive preprocessing, and achieving robust and efficient reconstructions.

The aforementioned methods differ primarily in their utilisation of sensor data in UCT. For instance, refs. [17,18] employ both sound-speed and reflection images reconstructed from raw measurements, while other approaches rely on transformed or intermediate representations. Specifically, refs. [19,20,22] extract time-of-flight (ToF) features from RF signals, and [21] utilizes a paraxial approximation to model wave propagation before feeding the resulting wavefield into a neural network. These approaches offer different trade-offs between physical interpretability, computational efficiency, and input representation.

In contrast, methods such as [23,24] aim to directly leverage raw RF data. The untrained-network FWI framework [23] integrates the acoustic forward model within an optimization process, while DeepUCT [24] learns a direct mapping from time-series sensor data to spatial images using supervised learning. These distinctions highlight the diversity of design choices in UCT deep learning, ranging from physics-informed optimization to fully data-driven approaches.

Inspired by DeepUCT [24], we propose the Multi-Channel Transducer Network (CUCT-Net), a convolutional neural network designed to address fundamental limitations of conventional FWI when applied to high-contrast tissues. In practical UCT settings, tissues such as bone exhibit large SoS contrasts that challenge iterative inversion methods [1], particularly under limited transducer counts and in the absence of reliable initial guesses.

Building on a direct signal-to-image learning paradigm, CUCT-Net introduces a multi-input feature encoding strategy, in which raw measurements from individual transducers are processed independently and subsequently fused with SU to enhance feature extraction under sparse sensing conditions.

Rather than pursuing continuous physical parameter inversion, this work presents a proof of concept for direct SoS estimation over a quantized value space and for direct tissue segmentation from raw ultrasound signals, both operating in reduced-sensor UCT configurations. Here, ‘quantized’ refers to discretization of the SoS value into a finite set of admissible levels (not spatial discretization due to pixelization). In this study, the SoS reconstruction is formulated using a limited number of quantization levels selected to span a broad, physically plausible SoS range. This design choice is deliberate: for high-contrast UCT scenarios, the quantization is defined with relatively large step sizes to enable robust discrimination of strong acoustic contrasts. For the brain tissue experiments, the eight output classes correspond to major tissue categories of practical relevance. This formulation allows stable learning and robust inference under conditions where conventional inversion methods are known to be unstable.

The proposed architecture operates directly on raw sensor data without preprocessing and is designed for computational efficiency and flexibility across different UCT system setups. The key contributions of this work are summarized as follows:

- No Dependency on Initial Guess: Unlike traditional FWI methods, the proposed deep learning approach does not rely on a solid initial guess and effectively handles high-contrast SoS regions, overcoming a significant limitation of FWI techniques.
- Direct Sensor Data Processing: In comparison to most neural networks, CUCT-Net is among the few models that directly process raw sensor data to generate UCT images, eliminating the need for preprocessing steps such as generating ToF images and reducing computational overhead.
- Modular Architecture with Enhanced Adaptability: CUCT-Net introduces a modular design that mirrors the UCT mechanism, allowing adaptation to different UCT setups by adjusting the number of input networks. The design delivers improved qualitative image fidelity with clearer boundaries, more accurate shapes, and fewer artifacts, supporting robust performance in complex SoS regions.
- Sensor-Aligned Multi-Encoder Architecture with Shift Units: The proposed model introduces a multi-encoder design tailored to the multi-transducer UCT acquisition process, enabling structured processing of sensor-specific signals. Building upon this architecture, Shift Units are incorporated to enhance feature extraction by capturing localized variations within each encoder stream, improving representation capability under complex wave propagation conditions.

This framework enables end-to-end quantized SoS estimation and tissue-level segmentation directly from raw UCT measurements. To the best of our knowledge, the proposed approach introduces three fundamental contributions. First, it presents a sensor-aligned multi-encoder architecture that preserves the distributed structure of multi-transducer UCT acquisition, rather than collapsing all receiver signals into a single representation. Second, it formulates UCT as a discretized prediction problem by quantizing the sound-speed space, transforming the conventional continuous inverse reconstruction into a classification-based objective that improves stability in ill-posed and high-contrast scenarios. Third, it establishes a direct signal-to-segmentation paradigm, learning tissue-level representations directly from raw sensor data without intermediate reconstruction. These contributions fundamentally distinguish the proposed method from existing approaches that primarily focus on continuous inverse reconstruction or rely on intermediate representations. In contrast, the Shift Units and transfer learning strategy serve as architectural refinements that further enhance performance within this framework. Together, these elements motivate the development of data-driven UCT pipelines that reduce reliance on iterative inversion under limited measurements.

## 2. Materials and Methods

This section provides a comprehensive overview of the experiments conducted, covering data collection, CUCT-Net architecture, loss function, evaluation metrics, training parameters, and computational hardware specifications.

### 2.1. Data Generation and Datasets

**Figure 1:**
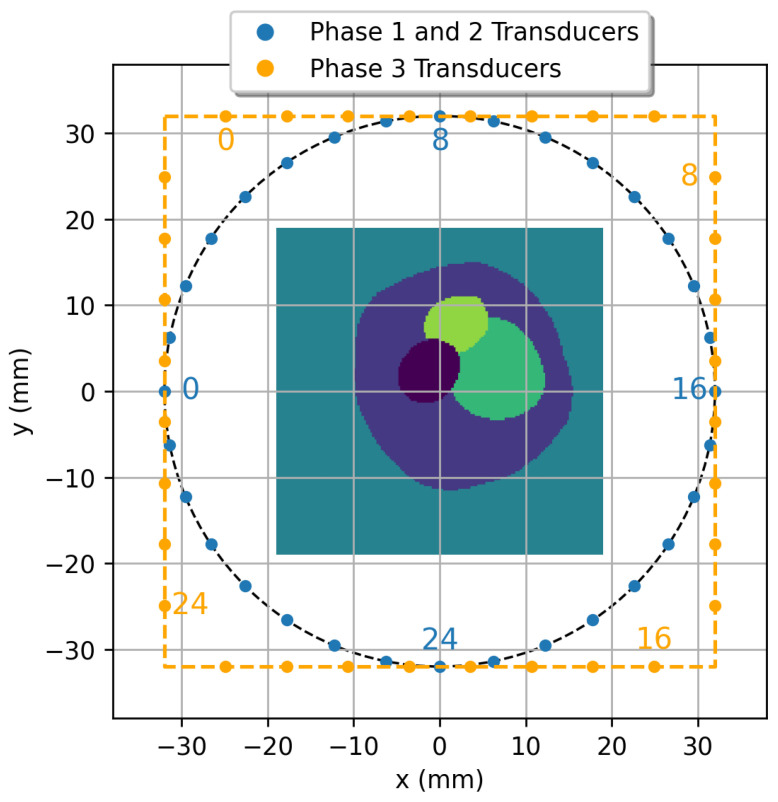
The simulation setup featured a centrally positioned square ROI surrounded by a ring of 32 transducers arranged clockwise. Two transducer configurations are highlighted: blue transducers represent Phase 1 and 2 experiments, whereas orange transducers correspond to Phase 3 experiments. Within the central phantom, different colors indicate distinct SoS values assigned to each region.

Data acquisition and analysis were performed using the k-Wave Toolbox v1.4 [25], a MATLAB (R2025B) library for simulating acoustic wave propagation. The experimental setup consists of 32 transducers arranged in either a uniform circular or square configuration surrounding the focal region (Figure 1). The circular configuration has a diameter of 64 mm, corresponding to 256 pixels at a spatial resolution of 0.25 mm per pixel, while the square configuration has a side length of 64 mm. Each transducer functions as both an emitter and a receiver.

The region of interest (ROI) is positioned at the center of the transducer array and spans 153×153 pixels, corresponding to a physical area of 38×38 mm^2^. For signal generation, the transducers emit a broadband Ricker-like pulse with a dominant frequency of 0.8 MHz and a temporal threshold of 3.2 µs. The effective bandwidth ranges approximately from 0.4 to 1.2 MHz, determining the achievable spatial resolution and penetration depth in the simulated imaging domain. This frequency range is consistent with clinical UCT systems such as SoftVue [26] and QT [27]. Data were recorded over 1000 effective time steps plus the initial reference time point (1001 total samples) per acquisition cycle with a temporal resolution of 0.1 µs. During acquisition, the transducers operate in a round-robin sequence: one transducer emits while the remaining 31 act as receivers. This emission–reception process constitutes one acquisition cycle, and 32 cycles were performed to collect sensor data for each phantom.

Two data sources were used in the experiments: synthetic disc-based phantoms inspired by the Shepp–Logan model [28], and anatomical brain phantoms derived from the Distorted Brain Benchmark (DBB) [29]. For the disc phantoms, three variants—Original, Distorted, and Mixed—were defined to evaluate robustness under structural perturbations, and experiments were conducted using both circular and square sensor geometries. The DBB-derived phantoms were employed to assess generalization to anatomically realistic brain structures.

#### 2.1.1. Synthetic Disc Phantoms (Shepp–Logan-Inspired)

For each synthetic disc phantom, a primary large disc was generated to define the ROI. One to four smaller discs were then randomly instantiated within this region, with radii randomly sampled within a predefined range. The phantom was surrounded by water with a fixed speed of sound (SoS) of 1500 $\;m\cdot s^{-1}$. To suppress artificial boundary reflections during wave propagation, perfectly matched layers (PML) [30] of 20 pixels were padded outside the transducer region.

Each ROI contains multiple discs assigned one of eight distinctive SoS values randomly selected between 900 and 2100 $\;m\cdot s^{-1}$. High-contrast regions (SoS differences exceeding 100 $\;m\cdot s^{-1}$) were intentionally introduced to emulate clinically relevant challenges, such as strong impedance discontinuities observed in brain and musculoskeletal imaging [1,31]. Such contrasts are known to induce cycle-skipping and local minima in FWI-based reconstruction [32].

**Figure 2:**
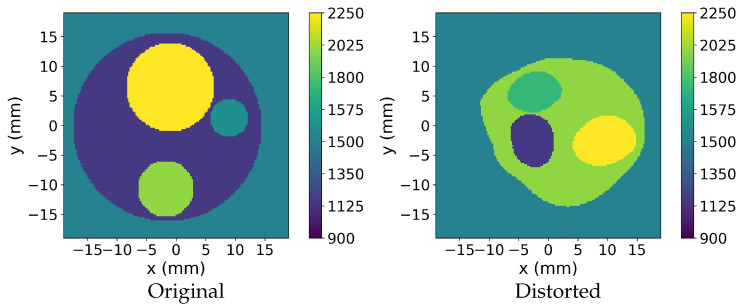
Simulated Shepp Logan Original and Distorted samples.

To increase structural diversity, random grid distortions were applied to the disc geometries, yielding both regular and perturbed shapes, as illustrated in Figure 2. From these configurations, three dataset variants were defined: Original, consisting of regular disc geometries without distortion; Distorted, containing only distorted samples; and Mixed, comprising equal proportions of randomly selected Original and Distorted samples.

#### 2.1.2. Dbb-Derived Anatomical Brain Phantoms

**Figure 3:**
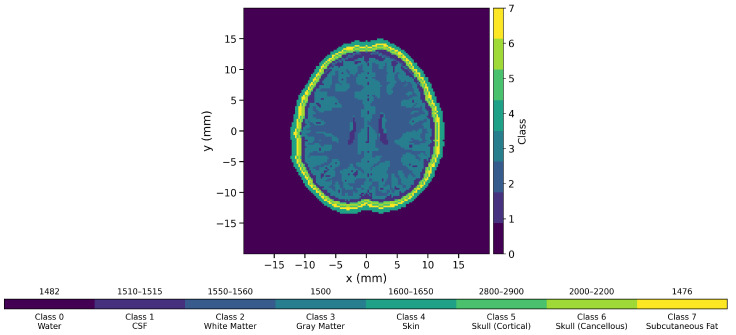
Class-based visualization of the DBB tissue segmentation map. The top panel shows the class map, while the bottom panel illustrates the corresponding SoS value ranges associated with each class.

To evaluate generalization under anatomically realistic and continuously varying acoustic properties, experiments were conducted using the DBB dataset, a publicly available 3D brain tissue segmentation dataset derived from T1-weighted MRI scans [29]. The original DBB ground-truth annotations consist of three primary brain tissue classes: cerebrospinal fluid (CSF), white matter, and gray matter. Two-dimensional slices were extracted from the 3D segmentations to construct planar phantoms. To better approximate a realistic UCT imaging scenario, additional surrounding layers were generated and appended to the brain region. These layers, created in our pipeline, represent skin, fat, and skull structures (including cortical bone and ventricular boundaries), thereby extending the anatomical model beyond the original DBB annotations. A representative DBB-derived phantom is shown in Figure 3, illustrating the overall anatomical structure and the spatial distribution of tissue classes. The brain region consists of multiple intracranial tissues, including CSF, white matter, and gray matter, while the surrounding layers model the scalp, subcutaneous fat, and skull (cortical and cancellous). These outer layers introduce strong acoustic contrasts, particularly at the skull boundary, which is critical for realistic ultrasound wave propagation. In contrast, several intracranial tissues exhibit relatively low acoustic contrast, such as the subtle differences between CSF, gray matter, and white matter, making their boundaries inherently difficult to distinguish. This combination of high-contrast outer structures and low-contrast internal regions creates a challenging segmentation scenario. The accompanying color bar provides a mapping between class indices, visualization colors, and their corresponding SoS values or ranges. This mapping serves as a reference for interpreting segmentation outputs, where each predicted class corresponds to a discrete tissue type with a predefined acoustic property.

Each tissue class (white matter, gray matter, cerebrospinal fluid, and additional outer layers, forming eight classes in total) was assigned a physiologically plausible SoS range according to the IT’IS Tissue Property Database [33]. Pixel-wise SoS values were randomly sampled within these ranges to generate spatially heterogeneous continuous SoS distributions. Acoustic forward simulations were then performed on these continuous maps to produce raw ultrasound sensor data. For training and evaluation, the continuous SoS values were discretized into their corresponding tissue classes, and predictions were compared against the binned ground-truth labels using segmentation metrics. All DBB phantoms were resized to 153×153 pixels, and the sensor configuration remained identical to that used in disc-based experiments to ensure consistent input–output dimensions.

#### 2.1.3. Experiment Setups

The training, validation, and testing splits for each experiment are summarized in Table 1. The experimental framework consists of three phases.

Phase 1 establishes baseline performance by training the network on clean synthetic disc-based phantoms without added noise. In this phase, 18,000/2000/2000 samples were used for training/validation/testing.

Phase 2 evaluates robustness under noisy measurement conditions using transfer learning initialized from the Phase 1 weights. Three disc-based dataset variants were considered: Original, Distorted, and Mixed. For each variant, additive white Gaussian noise was introduced to the simulated ultrasound signals at three SNR levels (20, 30, and 40) using the addNoise function from the k-Wave toolbox. This resulted in nine independent experimental settings (3 dataset variants × 3 SNR levels). For each setting, the data were divided into 500/250/250 training, validation, and testing samples.

Phase 3 assesses adaptability under new configurations and increased structural complexity. Transfer learning was again applied using the Phase 1 pre-trained weights. Two experiments were conducted: (i) Square, featuring a square sensor arrangement with disc-based phantoms, comprising 8000/1000/1000 training/validation/testing samples; and (ii) DBB, based on anatomical brain phantoms, comprising 18,000/2000/2000 training/validation/testing samples. Detailed splits are provided in Table 1 (rows 3–4).

Across all phases, the input sensor data were represented as a tensor of size 32×1001×32 corresponding to 32 transmitters, 1 initial plus 1000 time steps, and 32 receivers. The output SoS maps have dimensions of 153×153 in size, matching the ROI and excluding the surrounding sensor and PML regions.

### 2.2. CUCT-Net Architecture

**Figure 4:**
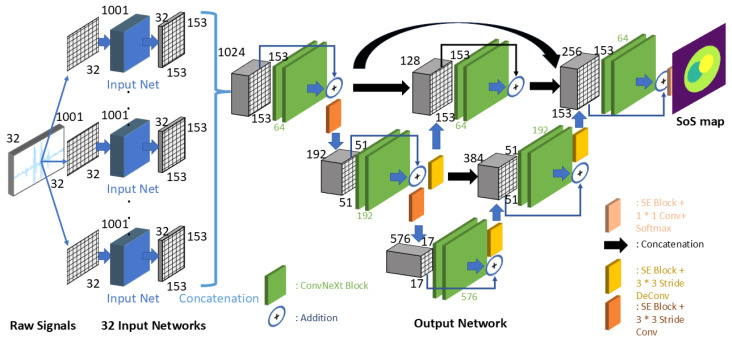
The figure showcases the pipeline of the proposed architecture. The neural network is divided into two main sections. The first section comprises 32 input networks, with each network dedicated to processing data recorded by a single receiver. The output feature maps from these networks are then concatenated to form a single input tensor for the second section of the architecture. This second section utilizes an enhanced UNet++ framework, integrating ConvNeXt and Squeeze-and-Excitation blocks to optimize performance.

The designed CUCT-Net processes the input data to cater to the simulation settings involving 32 transducers. The input data, initially in the format of *receivers* × *timesteps* × *transducers*, were segmented into 32 separate input tensors, each with dimensions of *timesteps* × *transducers*. The overall structure is shown in Figure 4. In the model architecture, 32 input networks, all sharing identical weights during the training phase, independently processed the 32 tensors. Each network produced its own unique set of final output feature maps. Subsequently, these feature maps were concatenated and fed into a secondary output network.

#### 2.2.1. Building Blocks

The foundational element of our designed network was modeled after the base block of the ConvNeXt architecture [34]. ConvNeXt represents a modern iteration of the traditional CNN design influenced by advancements in the transformer models. This was developed as an evolution of highly successful Vision Transformers [35] and ResNets [36] to combine the strengths of both architectures. For a ConvNeXt block, the input tensor is processed through a 7×7-depth-wise 2D convolution. This was followed by a linear layer that expanded the number of filters fourfold. Instead of using the Gaussian Error Linear Unit (GELU) [37], Scaled Exponential Linear Units (SELU) [38] are employed as activation functions, providing an enhanced capability to handle negative recorded signals. Subsequently, another linear layer acted on the feature maps, thereby reducing the number of filters to match the size of the inputs. Additionally, a skip connection is incorporated to retain more spatial information throughout the process.

Due to the heavy usage of concatenation throughout the entire network, we adopted a Squeeze-and-Excitation block [39] (SE block) to recalibrate the feature maps. The SE block is an architectural enhancement of CNNs designed to improve the representational power of a network by focusing on the channel-wise relationships of the features. It operates in two stages: the squeeze stage, which performs global average pooling to condense each channel of the feature map into a single value, effectively summarizing the global context of the channel; and the excitation stage, which uses a fully connected network to learn a set of channel-specific weights. These learned weights are then used to scale the original feature map, allowing the network to emphasize more essential channels and suppress less relevant ones. In our proposed network, SE blocks are strategically implemented following concatenation layers or skip connections to enable a more effective selection and utilization of feature maps.

**Figure 5:**
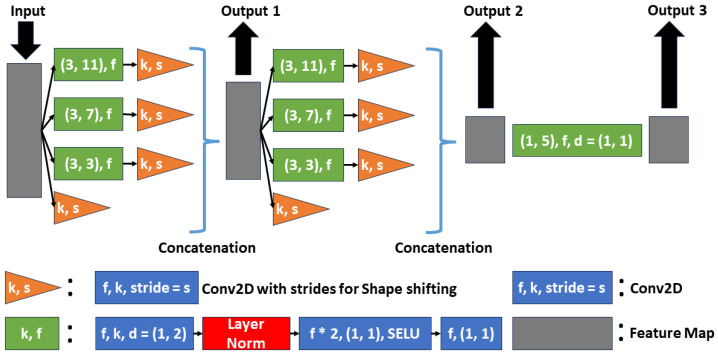
The SU of the model is divided into three stages and characterized by parameters such as the kernel size (k), number of filters (f), strides (s), and dilation rate (d). In the first two stages, the feature map passes through three paths, each consisting of an adapted ConvNeXt block with a dilated 2D convolution of varying kernel sizes. The final stage processes the feature map using a new, nondilated kernel size to enhance feature extraction.

A key element of our system is the SU, which is specifically designed to transform the dimensions of the input transducer signals into those of the output UCT image. Both a shape modifier and pooling mechanism are employed in the design of the SU, as illustrated in Figure 5. This component consists of three distinct stages: the first two are dedicated to altering the shape of the data, whereas the final stage further processes the reshaped feature maps. This structure allows for multiple outputs, facilitating a more detailed and thorough feature-extraction process.

The core of each stage is a base block (colored green in Figure 4 and Figure 5), built on the ConvNeXt block framework. However, in this design, depth-wise convolution layers typical of ConvNeXt blocks are replaced with 2D convolution layers featuring dilation rates [40]. This dilated convolution expands the kernel by inserting zeros among the nonzero kernel values and enhances the receptive field, capturing a more global context without compromising the resolution.

In the first two stages, shape shifting is achieved through an additional 2D convolution layer (colored orange) following the base block, employing strides to reduce the input size. Each stage contains three paths, each with a base block having a unique kernel size, facilitating comprehensive pooling. The input data also passes through a shape-shifting 2D convolution and is concatenated to the output of the stage, serving as a skip connection. The final stage processed the data without altering its shape. It introduces a base block with a new kernel size, which is different from the preceding stages, to provide a new field of view. At each SU stage, three outputs were produced for additional processing, ensuring dynamic transformation of the input data.

#### 2.2.2. Input Networks

**Figure 6:**
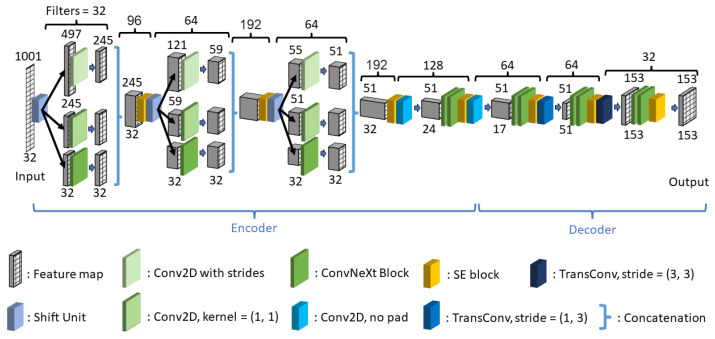
Illustration of the proposed Input Network: The diagram shows the number of filters at the top and the dimensions of each feature map along the sides. This network adopts an encoder–decoder structure, where the encoder reduces the data size and increases the filter count. The decoder inversely expands the data size while reducing the filters. The first three stages of the encoder utilize SUs, whereas the subsequent stages employ 2D convolution to reshape the data.

The complete structure of the input network is shown in Figure 6. At its core, this network acts as a mapping model that transforms the data received by each receiver into the view of the final UCT image. The network is divided into two parts: the encoder on the left and the decoder on the right, maintaining a conventional design in which the encoder reduces the data size in exchange for an increased number of filters.

Within the five stages of the encoder, the initial three utilize SUs to retain more features, offsetting the reduction in data size. The final two stages of the encoder adopt a more straightforward design owing to minor data size changes, employing 2D convolutions without padding. This approach naturally diminishes data dimensions through careful kernel size selection. Avoiding SU in these stages is a deliberate choice for enhancing memory efficiency. Using two transposed convolution layers, the feature maps of the decoder were expanded back to the dimensions of the predicted UCT images.

#### 2.2.3. Output Network

The output network received the combined feature map outputs from all 32 input networks. Because the input network outputs can be converted into individual UCT image predictions, the output network synthesizes the perspectives from all input networks (receivers) and produces a consolidated, fused SoS distribution image.

The design of the output network was based on the UNet++ architecture [41]. UNet++ is an advanced architecture that builds on the original U-Net [42] design, which is widely recognized for its effectiveness in medical image segmentation tasks. The key innovation of UNet++ is its redesigned skip pathways and nested dense convolutional block structures. Unlike the classic UNet, which has direct skip connections between the corresponding layers of the encoder and decoder, UNet++ introduces a series of intermediate convolutional layers into the skip pathways. This structure forms a dense grid of varying resolution feature maps, enhancing the information flow and gradients throughout the network.

In CUCT-Net’s adaptation of the output network, the full UNet++ architecture is modified to include only three layers, optimizing it for a more efficient downsampling process and better memory management. Diverging from the typical two-fold downsampling used in UNet++ and U-Net, a downsampling scale of 3 was chosen. This approach effectively accommodates 153×153 data dimensions, thereby preventing undue truncation. To compensate for this larger downsampling scale, the number of filters in the network was increased threefold after each downsampling step. Additionally, enhancements were made to the base blocks of the standard UNet++ design. The output network incorporates ConvNeXt blocks as processing elements and integrates SE blocks to improve the feature selection and utilization. Skip connections were integrated within each block of the output network to facilitate a smoother gradient flow throughout the layers. The entire network concludes with a Softmax activation layer, which is used to predict the probability of each SoS class.

The overall architecture of the network is represented succinctly by Equation (1), where $F_{input}$ symbolizes the input network, and $G_{output}$ represents the output network. The equation is defined as follows:

(1) $$\begin{array}{c} X^{\prime }=G_{output}\left(\sum_{i=1}^{32}F_{input}\left(X_{i}\right)\right) \end{array}$$

$X^{\prime }$ indicates the final prediction output of the network, $X_{i}$ denotes the ultrasound signal received from the $i^{th}$ receiver, and the summation symbol ∑ denotes the concatenation of the outputs of the input networks before they are fed into the output network.

### 2.3. Loss Function and Metrics

To optimize the network during training, a combined loss function composed of focal loss [43] and Dice loss is employed.

(2) $$\begin{array}{c} L_{\mathrm{combined}}=L_{\mathrm{focal}}+L_{\mathrm{dice}} \end{array}$$

Focal loss is a modified cross-entropy loss function designed to address class imbalance by emphasizing hard, misclassified examples. It is defined as:

(3) $$\begin{array}{c} L_{\mathrm{focal}}\left(p_{t}\right)=-\alpha_{t}{\left(1-p_{t}\right)}^{\gamma }\log\left(p_{t}\right) \end{array}$$

Here, $p_{t}$ denotes the predicted probability of the ground-truth class, γ is the focusing parameter, and $\alpha_{t}$ is a class-balancing factor. Dice loss is a region-based loss function that directly optimizes the overlap between the predicted segmentation and the ground truth. It is defined as:

(4) $$\begin{array}{c} L_{\mathrm{dice}}=1-\frac{2\sum_{i}p_{i}g_{i}+\epsilon }{\sum_{i}p_{i}+\sum_{i}g_{i}+\epsilon } \end{array}$$

where $p_{i}$ and $g_{i}$ denote the predicted probability and ground-truth label of pixel *i*, respectively, and ϵ is a small constant for numerical stability, set to be $10^{-5}$ during training.

Model performance is evaluated using multiple quantitative metrics, including Accuracy, Dice coefficient, Intersection-over-Union (IoU), and Structural Similarity Index Measure (SSIM). Accuracy is defined as the proportion of pixels for which the predicted label matches the ground truth. Beyond pixel-wise accuracy, the Dice coefficient is employed to assess region-level similarity between the predicted segmentation and the ground truth. It is defined as:

(5) $$\begin{array}{c} Dice\left(X,Y\right)=\frac{2|X\cap Y|}{\left|X|+|Y\right|} \end{array}$$

where *X* and *Y* denote the ground-truth and predicted segmentations, respectively. The Dice coefficient ranges from 0 to 1, with higher values indicating greater overlap between the predicted segmentation and the ground truth. To further assess segmentation performance, the IoU metric is introduced.

(6) $$\begin{array}{c} IoU\left(X,Y\right)=\frac{\left|X\cap Y\right|}{\left|X\cup Y\right|} \end{array}$$

IoU ranges from 0 to 1 and offers a measure similar to the Dice coefficient, while imposing stronger penalties for pixel-wise mismatches, thereby serving as a stricter and more informative metric. The SSIM is used to evaluate the similarity between the generated and ground-truth SoS distribution images. SSIM assesses image similarity by comparing structural information, luminance, and contrast. It is defined as:

(7) $$\begin{array}{c} SSIM\left(X,Y\right)=\frac{\left(2\mu_{x}\mu_{y}+c_{1}\right)\left(2\sigma_{xy}+c_{2}\right)}{\left(\mu_{x}^{2}+\mu_{y}^{2}+c_{1}\right)\left(\sigma_{x}^{2}+\sigma_{y}^{2}+c_{2}\right)} \end{array}$$

where $\mu_{x}$ and $\mu_{y}$ denote the mean intensities of images *X* and *Y*, respectively, $\sigma_{x}^{2}$ and $\sigma_{y}^{2}$ are the corresponding variances, $\sigma_{xy}$ is the covariance between *X* and *Y*, and $c_{1}$ and $c_{2}$ are constants used to stabilize the division. SSIM values range between −1 and 1, where 1 indicates perfect similarity, 0 indicates no similarity, and −1 indicates perfect anti-correlation.

### 2.4. Training Details

The dataset includes eight discrete SoS values ranging from 900 to 2250 $\;m\cdot s^{-1}$, with increments of 225 $\;m\cdot s^{-1}$ between each value, while the surrounding water is set to 1500 $\;m\cdot s^{-1}$. In the preprocessing stage, these SoS values were assigned specific labels before being fed into the neural network. Label 0 is designated for the water surrounding the phantom, and the subsequent labels represent the other seven SoS values in ascending order. The data were then transformed into a one-hot encoding format, enabling the neural network to output probabilities for each of the eight SoS values. The class with the highest probability is selected to generate the final UCT image. After determining the chosen SoS value, the final prediction is visualized and stored as a heat map created using the Matshow function provided by Matplotlib 3.6.3 [44].

The architecture was built on Keras [45] using TensorFlow [46] as the backend for implementation. The convolution layers were initialized using the Kaiming Normal method [47] for faster convergence. Optimization during the tests was conducted using the Adam optimizer [48] over 100 epochs. A grid search determined the optimal learning rate: 8 × 10^−5^ for the Original dataset and 5 × 10^−5^ for the Distorted and Mixed dataset. Given the sizeable 3D input transducer signals, a smaller batch size of 10 was used for all experiments. During training, a single-precision floating-point format was applied to all input tensors to reduce the memory usage.

For a consistent comparison, all the deep learning models in Phase 1 were trained for 100 epochs with an early stopping mechanism to prevent overfitting. Most models converged within the first 20 epochs and showed steady improvement thereafter. Phases 2 and 3 focused on transfer learning, allowing the proposed CUCT-Net model to adapt to different sensor data. The transfer learning datasets and training cycles were intentionally kept small to facilitate rapid adaptation while maintaining the performance. In Phase 2, transfer learning was performed on noisy data for five epochs using the same learning rate as in Phase 1 for each dataset. In Phase 3, the square sensor formation dataset was trained for 10 epochs, whereas the DBB dataset was trained for 50 epochs with a learning rate of 5 × 10^−5^. All training was conducted on an NVIDIA A100 GPU with 80 GB of VRAM to ensure efficient and reliable processing.

## 3. Results

The performance of the models was assessed using four metrics: the Dice coefficient, Accuracy, IoU, and SSIM. Collectively, these metrics provide a comprehensive evaluation of model performance by capturing the spatial accuracy and structural fidelity, ensuring that the segmentation quality aligns closely with the ground truth data across different dimensions. The model was modified into three distinct versions to reduce memory requirements and to support an ablation study by isolating the contributions of key architectural components: CUCT-Net Small, Base, and Large. CUCT-Net Large retains the comprehensive design outlined in Section 2.2. The CUCT-Net Base reduces memory usage by excluding SUs from the input networks. Finally, CUCT-Net Small further minimizes memory demands by replacing double ConvNeXt blocks in both input and output networks with single ConvNeXt blocks, significantly lowering the total parameter count.

FWI is implemented as a comparison method using the open-source Stride library [49]. The inversion followed a frequency continuation strategy consisting of five frequency blocks with maximum frequencies of 0.1, 0.3, 0.5, 0.7, and 0.8 MHz. Each frequency block is optimized for 20 iterations, and at each iteration, a subset of 16 shots is randomly selected to balance the computational efficiency and robustness. The inversion is initialized from a homogeneous SoS model of 1500 $\;m\cdot s^{-1}$, without using prior structural information.

The forward wave propagation in Stride is solved using the IsoAcousticDevito operator with the OT2 kernel, which corresponds to a second-order accurate time discretization of the acoustic wave equation and is implemented via Devito [50]. To improve the numerical accuracy of the iterative inversion baseline, FWI experiments are conducted on a higher spatial resolution grid of 256×256 compared with the learning-based pipeline. Because FWI requires forward data that are consistent with the discretization, solver, and grid used during inversion, the corresponding sensor measurements are re-simulated within Stride on the same grid and temporal sampling. In contrast, the learning-based methods operate on k-Wave simulated data as described earlier.

The inversion is performed using gradient descent with a fixed step size of 2.0. Gradients are aggregated using a global gradient processing strategy, and the model parameters are constrained to a physically plausible range of [800, 2400] $\;m\cdot s^{-1}$ during optimization. The stopping criterion is defined by a fixed iteration budget determined by the number of frequency blocks and the number of iterations per block. Due to the high computational cost of FWI, the reported FWI metrics are computed as averages over 20 randomly selected test samples.

For segmentation-based evaluation metrics, including Accuracy, Dice coefficient, and IoU, the continuous FWI outputs are mapped to the nearest discrete SoS values to ensure consistency with categorical ground truth labels. The SSIM is computed directly using continuous FWI reconstructions without discretization. This evaluation protocol ensures a consistent and unbiased comparison across methods.

The DeepUCT model [24] was chosen for comparison owing to its similar data format and transducer setup. DeepUCT utilizes a dual-structure model consisting of convolution layers with varying stride and padding sizes for shape mapping between the input signal and output feature map. The model was benchmarked against Full Waveform Inversion with the Source Encoding (FWI-SE) [51], exhibiting high performance in both accuracy and efficiency compared to traditional FWI methods. To modify the DeepUCT model for multiclass prediction, its final layer is altered to include a Softmax activation layer. In addition, the loss function was switched to the previously mentioned combined loss function.

Table 2 provides an overview of the weight size, parameter count, and prediction time for the four evaluated neural networks, along with the prediction time of FWI for comparison. In the proposed model, the 32 input networks utilize identical weights due to their similar functionality. This shared-weight strategy reduces the model’s overall size and parameter count, resulting in a more compact architecture. However, the inclusion of 32 input networks increases prediction times compared to DeepUCT.

In terms of computational complexity, the number of floating point operations (FLOPs) is reported for each model. CUCT-Small (340.3 GFLOPs) is slightly more efficient than DeepUCT (377.9 GFLOPs), while CUCT-Base (547.5 GFLOPs) and CUCT-Large (1347.8 GFLOPs) provide higher-capacity configurations with increased computational cost, demonstrating a flexible trade-off between efficiency and performance. Despite its lower parameter count and GFLOPs, CUCT-Small exhibits a longer inference time than DeepUCT. This behavior is primarily attributed to the multi-branch architecture, where 32 encoder streams introduce additional overhead in memory access and kernel scheduling, as well as the use of custom modules. Consequently, inference time is influenced not only by model size or FLOPs but also by architectural design and implementation efficiency.

The full version, outlined in Section II-B, is designated as CUCT-Net Large. This model incorporated 32 input networks and a dual ConvNeXt block design for enhanced processing. The Large model has 21,292,904 parameters, a weight size of 245 MB, and an image prediction time of 0.73 s per image. To analyze the contribution of each architectural component, the following model variants were introduced as part of an ablation study, each systematically removing or modifying key structures from the proposed network to assess their impact on performance. The second variant, CUCT-Net Base, alters the CUCT-Net Large configuration by replacing SUs with a single convolution layer, aiming for quicker processing and reduced memory requirements. The Base model comprised 15,360,264 parameters, a weight size of 177 MB, and a prediction time of 0.28 s. This model has 27.8% fewer parameters, is 27.9% smaller in weight size, and is 2.61 times faster than the large model. The final variant, CUCT-Net Small, further downsizes the design by limiting the SU and maintaining a single ConvNeXt block in sections where CUCT-Net Large and Base employ double ConvNeXt blocks. The Small model adheres to the core design principle of 32 input networks and one output network despite having fewer convolution layers. It possesses 11,648,200 parameters, a weight size of 134 MB, and a prediction time of 0.23 s. In comparison to the Large model, the Small variant shows a 45.3% reduction in both parameters and weight size and is 3.17 times faster than CUCT-Net Large.

**Figure 7:**
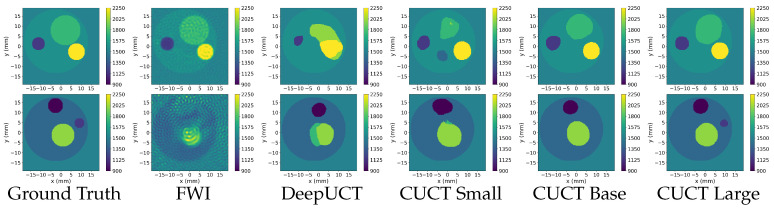
This figure presents a comparative analysis of the results from various models tested using the Original dataset. The first column shows the ground-truth UCT images. FWI results are shown in the second column. The predictions from DeepUCT occupy the third column, whereas the fourth column shows the results from CUCT-Net Small. The fifth column contains the outputs from the CUCT-Net Base, and the sixth column shows the predictions by CUCT-Net Large. The comparison revealed that CUCT-Net Large produced the most accurate predictions among all tested methods.

**Figure 8:**
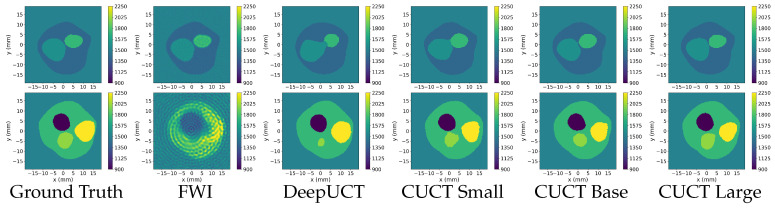
This figure presents a comparative analysis of the results from the various models tested using the Distorted dataset. The first column displays the ground-truth UCT images. FWI’s result is located in the second column. Predictions from DeepUCT occupy the third column, whereas the fourth column shows the results from CUCT-Net Small. The fifth column contains the outputs from the CUCT-Net Base, and the sixth column shows the predictions by CUCT-Net Large. The comparison revealed that CUCT-Net Large produced the most accurate predictions among all methods tested.

**Figure 9:**
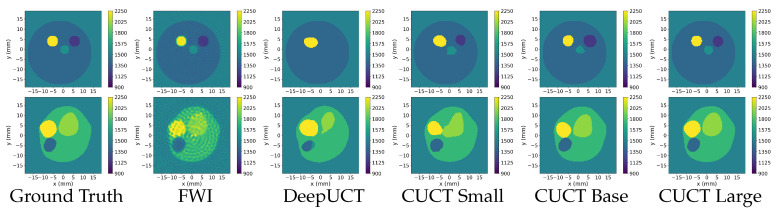
This figure presents a comparative analysis of the results from various models tested using the Mixed dataset. The first column displays the ground-truth UCT images. FWI’s result is located in the second column. Predictions from DeepUCT occupy the third column, whereas the fourth column shows the results from CUCT-Net Small. The fifth column contains the outputs from the CUCT-Net Base, and the sixth column shows the predictions by CUCT-Net Large. The comparison revealed that CUCT-Net Large produced the most accurate predictions among all methods tested.

**Figure 10:**
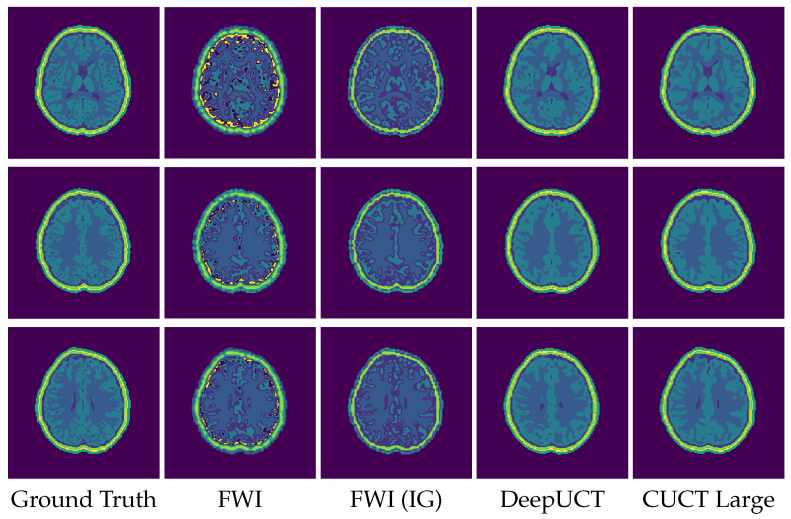
This The figure compares the performance of different methods on the DBB dataset, where different colors indicate distinct tissue types associated with specific SoS range. From left to right, the columns show the ground-truth UCT images, FWI results initialized with a uniform SoS value of 1500 m/s, FWI (IG) results initialized with the mean skull tissue SoS, DeepUCT predictions, and CUCT-Net Large predictions. Overall, CUCT-Net Large exhibits the closest agreement with the ground truth among the compared methods.

For a precise comparative analysis, consistent settings were maintained across all tested neural networks, including the combined loss, Adam optimizer, and Kaiming initialization. Each model was tuned to ensure an optimal performance. The following section describes the experiments conducted in all three phases. Section 3.1 (Phase 1) focuses on clean sensor data and includes tests on three datasets: the Original, Distorted, and Mixed. The metrics are presented in Table 3, Table 4 and Table 5, with the best performance highlighted in bold, whereas the results are shown in Figure 7, Figure 8 and Figure 9. Section 3.2 presents tests on noisy data from the Original, Distorted, and Mixed datasets under three signal-to-noise ratios (SNR) of 20, 30, and 40, with the metrics detailed in Table 6. Finally, Section 3.3 presents the Phase 3 experiments, including the Square sensor formation and the DBB phantom evaluation. Quantitative results for the Square dataset are summarized in Table 7, while performance metrics for the DBB dataset are reported in Table 8. Representative predictions for the DBB phantoms are shown in Figure 10.

### 3.1. Phase 1: Clean Sensor Data

#### 3.1.1. Original Dataset

Phase 1 results for the Original Dataset are summarized in Table 3. The tests show that FWI achieves a Dice coefficient of 0.5540, accuracy of 0.5329, and IoU of 0.2407. In comparison, the DeepUCT model achieved a significantly higher performance with a Dice coefficient of 0.9444, an accuracy of 0.9500, an IoU of 0.8611, and an SSIM of 0.9249. CUCT-Net Small further improved these metrics, achieving a Dice coefficient of 0.9664, accuracy of 0.9677, IoU of 0.9086, and SSIM of 0.9439. CUCT-Net Base performs even better, reaching a Dice coefficient of 0.9791, an accuracy of 0.9802, an IoU of 0.9418, and an SSIM of 0.9622. The highest performance was achieved by CUCT-Net Large, which attained a Dice coefficient of 0.9811, an accuracy of 0.9819, an IoU of 0.9456, and an SSIM of 0.9706.

Figure 7 presents the UCT images generated from the Original dataset, characterized by smaller minimum radii compared to Distorted data, which poses a challenge for predicting small inner discs. The first row depicts a phantom with a lower contrast between the discs and the surrounding water. FWI captures the overall structure and disc boundaries; however, its output is noisy and lacks smoothness. The neural networks face challenges in accurately separating class 5 ($1800\;m\cdot s^{-1}$) from the class 4 contour disc ($1575\;m\cdot s^{-1}$), as seen in areas where the boundaries blur. DeepUCT struggles to distinguish between the inner discs, resulting in merged predictions. In contrast, the CUCT-Net family demonstrated progressively better performance as the model size increased, with CUCT-Net Large achieving the most accurate prediction and closely matching the ground truth.

The second row shows UCT images with larger inner discs and a higher contrast than the surrounding regions. While all models successfully predicted the ROI contour, their ability to capture the smallest disc varied. FWI produced noisy outputs, and DeepUCT struggled with clear boundary delineation. Among the models, CUCT-Net Large provided the most accurate prediction and effectively captured the smallest disc.

#### 3.1.2. Distorted Dataset

The results of the experiments on the Distorted dataset are summarized in Table 4. FWI achieved a Dice coefficient of 0.6793, an accuracy of 0.6525, an IoU of 0.2755, and an SSIM of 0.4224. The DeepUCT model recorded a Dice coefficient of 0.9498, an accuracy of 0.9568, an IoU of 0.8446, and an SSIM of 0.9258. The CUCT-Net Small achieved 0.9664, 0.9775, 0.9169, and 0.9472 for Dice, accuracy, IoU, and SSIM, respectively. The CUCT-Net Base recorded 0.9819, 0.9833, 0.9377, and 0.9567 for these metrics, while CUCT-Net Large achieved the highest scores of 0.9876, 0.9881, 0.9468, and 0.9686, respectively.

Figure 8 illustrates the varied performances of models with different ROI shapes. The first row features a relatively simpler phantom, where all models perform well. FWI captures the general shape of the inner discs with high resemblance, but introduces significant noise. DeepUCT and CUCT-Net Small failed to differentiate between the two inner discs, merging them into a single region. In contrast, CUCT-Net Base and CUCT-Net Large produced highly accurate predictions, clearly separating the inner discs with minimal errors.

The phantom in the second row contained a low SoS disc that contrasted sharply with the surrounding higher-SoS regions. This high contrast causes blurred outcomes in FWI. Meanwhile, neural networks handle this contrast effectively, and all predictions accurately capture the low SoS disc. The differences among the neural network models lie in the prediction of the class 5 disc ($1800\;m\cdot s^{-1}$) and class 6 disc ($2025\;m\cdot s^{-1}$), where CUCT-Net Large successfully predicts the fine details of the class 6 disc.

#### 3.1.3. Mixed Datasets

The quantitative results for the Mixed dataset (see Table 1) are presented in Table 5. FWI achieved a Dice coefficient of 0.6197, accuracy of 0.5623, IoU of 0.2609, and SSIM of 0.4101. DeepUCT recorded 0.9484, 0.9555, 0.8621, and 0.9255 for Dice, accuracy, IoU, and SSIM, respectively. CUCT-Net Small achieves 0.9727, 0.9757, 0.9255, and 0.9458 for these metrics. The CUCT-Net Base yielded 0.9793, 0.9813, 0.9421, and 0.9546, while CUCT-Net Large achieved the highest scores of 0.9841, 0.9857, 0.9528, and 0.9712, respectively.

Figure 9 shows UCT images generated from the Mixed dataset. The first row shows a three-disc phantom with small discs. FWI identifies all discs, but introduces noise. DeepUCT predicted only one disc and missed other discs. CUCT-Net Small predicts all three discs but with some blurring and size inaccuracies. CUCT-Net Base improved the overall disc shapes and better captured the phantom’s structure; however, minor inaccuracies remained in one of the inner discs. CUCT-Net Large provides the most accurate prediction, closely matching the ground truth.

The second row shows a phantom with larger inner discs. FWI produces noisy reconstructions, obscuring finer details. DeepUCT correctly identifies the general structure, but struggles with accurate boundaries. CUCT-Net Small and Base improved the shape and boundaries of the discs, with the Base providing clearer contours. CUCT-Net Large delivered the most precise prediction, closely resembling the ground truth.

The experimental results across the Original, Distorted, and Mixed datasets demonstrate the superior performance of the CUCT-Net models compared with FWI and DeepUCT. FWI effectively identifies general structures, but introduces significant noise and lacks precision in disc boundaries. DeepUCT achieves better accuracy, but often struggles with low-contrast regions and small inner discs. Among the CUCT-Net models, Small consistently predicted all structures, but with minor blurring or size inaccuracies. The Base improves on these predictions with sharper boundaries and better overall structure, but occasionally falters in low-contrast regions. CUCT-Net Large consistently delivered the most accurate predictions, closely matching the ground truth across all datasets, with minimal errors in challenging areas.

### 3.2. Phase 2: Noisy Sensor Data

Phase 2 experiment further evaluates the ability of CUCT-Net Large to generalize to noisy data. The pretrained weights from Phase 1 were directly applied to each dataset, followed by transfer learning using 500 noisy data samples. CUCT-Net Large quickly adjusts to noisy data, with the transfer learning process requiring only 500 training samples, 2.8% of the data used in Phase 1’s full training process, and only five epochs. The quantitative results for the noisy Original, Distorted, and Mixed datasets are listed in Table 6.

FWI demonstrates consistent performance across all SNR levels in terms of classification metrics. However, it shows a clear decline in the SSIM as the SNR decreases, indicating that its ability to maintain spatial and intensity relationships is affected by noise. This discrepancy between the classification metrics and SSIM can be explained by the mapping process used to evaluate the classification metrics. To ensure a fair comparison, the continuous SoS outputs of FWI were mapped to the nearest discrete SoS values, aligning them with the categorical outputs required for classification metrics. While this ensures consistency, it can mitigate the true impact of noise on metrics such as Dice, Accuracy, and IoU. In contrast, SSIM directly evaluates spatial and intensity relationships using the raw output of the FWI method, making it more sensitive to noise and reflective of FWI’s underlying limitations in noisy environments. DeepUCT exhibits moderate robustness to noise, maintaining relatively high metrics, such as Dice and SSIM, across SNR levels. However, its performance degrades slightly at lower SNRs, particularly in IoU, indicating some sensitivity to increased noise levels. However, DeepUCT consistently outperformed FWI, achieving a significantly better prediction accuracy and higher reliability under noisy conditions.

CUCT-Net Large consistently delivered the best results across all SNR levels, with Dice scores (0.9738–0.9803) and SSIM values (0.9497–0.9678) remaining robust, even at the lowest SNRs. Its minimal performance degradation at an SNR of 20 highlights its adaptability and ability to process noisy sensor data effectively. Compared to FWI and DeepUCT, CUCT-Net Large achieves a significantly superior prediction quality, with more precise boundaries and more reliable representations of the ground truth.

In summary, FWI struggles significantly in noisy environments, producing noisy and inaccurate reconstruction. DeepUCT offers moderate robustness but is still affected by increased noise levels. CUCT-Net Large is the most robust and effective method, maintaining high accuracy and reliability across all SNR levels, making it the most suitable model for noisy conditions.

### 3.3. Phase 3: Generalization and Segmentation

Phase 3 experiments aim to further evaluate the generalization ability of CUCT-Net Large from two complementary perspectives: robustness to changes in sensor configuration and extension to direct tissue-level segmentation from raw UCT measurements. Specifically, the Square experiment tests adaptability to a different transducer arrangement, while the DBB experiment evaluates performance on tissue-derived brain phantoms with irregular and complex structures. In the Square experiment, the sensors were arranged in a square formation surrounding the phantom, as shown in Figure 1 (orange sensors), replacing the circular arrangement used in previous phases. DBB experiments are conducted by creating phantoms that mimic the structure and tissue properties of the human brain, where the sample phantom can be seen in Figure 3.

The Square and DBB experiments were conducted using transfer learning, with CUCT-Net Large initialized from Phase 1 pre-trained weights obtained from the Mixed dataset. Transfer learning substantially accelerated the convergence of the model in both cases. For the Square dataset, only 10 training epochs were required to achieve satisfactory performance, reflecting the strong adaptability of the model to changes in the sensor geometry. This behavior can be attributed to the modular design of the CUCT-Net Large, which enables the efficient reuse of learned representations when the sensor locations are modified. The DBB experiment posed greater challenges due to the increased structural complexity of the phantoms and the transition from quantized SoS classes to tissue-specific SoS ranges. Nevertheless, transfer learning provided a significant advantage: after loading Phase 1 pre-trained weights, the model reached a performance comparable to that of a from-scratch training after a single epoch, whereas approximately 30 epochs were required to attain a similar performance when trained without pre-training. Additional fine-tuning over 40 epochs further improved the accuracy, demonstrating that transfer learning not only reduces the training time but also stabilizes the optimization for complex phantom configurations.

The quantitative results of these experiments are summarized in Table 7 and Table 8. In the Square experiment, CUCT-Net Large achieves a Dice coefficient of 0.9804, accuracy of 0.9816, IoU of 0.9262, and SSIM of 0.9548, indicating that the proposed model maintains a high segmentation performance under an alternative transducer arrangement. For the DBB experiments, the FWI baseline is configured with an increased number of time steps in the forward simulation, from 1000 to 2000, to improve numerical accuracy. Two FWI setups are evaluated, both with the adapted constraint range of [1450, 3000] $m\cdot s^{-1}$: a standard FWI initialized with a uniform SoS value of 1500 $m\cdot s^{-1}$, and an FWI variant initialized with the provided skull layer as prior information, denoted as FWI (IG). The quantitative results are presented in Table 8. Under this configuration, the standard FWI achieves a Dice coefficient of 0.4126, which improves to 0.4901 with the informed initialization. DeepUCT further improves the performance, achieving a Dice coefficient of 0.7160±0.0459, accuracy of 0.8990±0.0152, IoU of 0.5815±0.0506, SSIM of 0.8257±0.0209, HD95 of 2.1677±0.2493, and Boundary F1 of 0.8595±0.0259. In comparison, CUCT-Net Large achieves the highest performance across all evaluated metrics, with a Dice coefficient of 0.8190±0.0325, accuracy of 0.9224±0.0135, IoU of 0.7104±0.0424, SSIM of 0.8651±0.0177, HD95 of 1.6831±0.1571, and Boundary F1 of 0.9074±0.0199. These results indicate consistently improved and stable segmentation performance on tissue-derived DBB phantoms. Compared to DeepUCT, CUCT-Net Large consistently improves all metrics, with notable gains in Dice, IoU, and Boundary F1, as well as a lower HD95, indicating more accurate segmentation and better boundary delineation. In addition, the reduced standard deviations across all metrics suggest more stable and consistent performance across samples.

**Figure 11:**
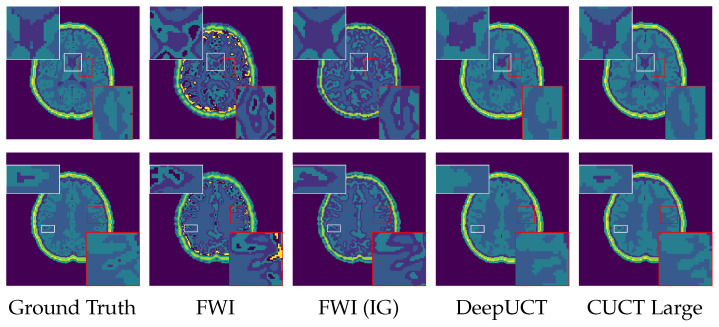
Zoomed -in views of DBB dataset samples highlighting fine structural details and boundary delineation across different methods, with improved agreement to the ground truth observed for CUCT-Net Large.

A qualitative comparison of the segmentation results is provided in Figure 10, with zoomed-in regions shown in Figure 11 to highlight boundary details. As observed, the proposed CUCT-Net produces the most accurate and consistent tissue boundaries, particularly at interfaces between adjacent structures. In contrast, the standard FWI exhibits noticeable boundary artifacts, especially near high-contrast regions, where it tends to introduce incorrect CSF predictions along the interfaces between white and gray matter. The informed initialization (FWI (IG)) partially mitigates these artifacts but still fails to accurately resolve fine structural details. DeepUCT further improves boundary smoothness and reduces noise; however, it tends to oversmooth tissue interfaces, resulting in the loss of finer anatomical details. Overall, CUCT-Net achieves a better balance between boundary accuracy and structural preservation, which is consistent with the improvements observed in boundary-sensitive metrics such as HD95 and Boundary F1.

Overall, the Phase 3 results demonstrate the robustness and adaptability of CUCT-Net Large across different sensor configurations and increasing phantom complexities. The model maintains strong performance under varying acquisition geometries and tissue-derived phantoms, supporting the feasibility of both quantized high-contrast SoS estimation and direct tissue segmentation from raw UCT sensor data. The results on the DBB dataset further indicate the model’s ability to generalize to irregular and complex brain tissue structures, with moderate reductions in Dice and IoU reflecting the increased structural and contrast variability relative to the simplified discretized training scenarios.

## 4. Discussion

This section analyzes the proposed CUCT-Net within the scope of direct inference from raw UCT sensor data, encompassing both quantized SoS estimation and tissue-level segmentation. The framework formulates UCT as a direct signal-to-image learning problem, in which quantized SoS classes or tissue labels are inferred directly from the measured ultrasound signals. Under this formulation, the network output represents either a quantized SoS map for high-contrast imaging scenarios or a tissue segmentation map for anatomically complex media. The following analysis discusses the experimental results obtained under these two problem settings and evaluates the robustness and generalization behavior of the proposed model accordingly.

There are two main types of errors in the prediction of UCT images. First, geometrical errors involve inaccuracies in the shape, size, or location of the SoS regions. This is evident in the distorted or misplaced discs observed in the predicted images, which are common challenges in UCT reconstruction. Even the best-performing models exhibit geometric errors due to inherent limitations in the inverse reconstruction process, such as limited angular sampling from discrete transducer arrangements, finite signal bandwidth restricting resolution, wave scattering and multipath propagation in heterogeneous media, and measurement noise. These factors contribute to reconstruction ambiguities and geometric inaccuracies, ultimately affecting key evaluation metrics including SSIM, Dice coefficient, and IoU.

Second, classification errors occur when the model predicts an incorrect SoS value for a given region. This can manifest as a failure to predict the correct SoS value or, in extreme cases, the inability to distinguish a region from its background of water. Classification errors are often caused by low contrast between regions or similarities in SoS values with neighboring areas. Neural networks may also prioritize larger, more distinct regions while neglecting smaller, low-contrast regions. In most instances, the incorrectly predicted SoS value is close to the ground truth, making it less noticeable in the SSIM scores. However, the Dice coefficient and IoU were more effective metrics for detecting these errors.

Although FWI predicts continuous SoS values directly, it is prone to weaknesses that limit its reliability. For geometrical errors, FWI performs well in low-contrast regions, but struggles in high-contrast areas, often distorting the ROI and producing results with lower quality. In terms of classification errors, FWI frequently generates oscillating SoS values in regions where the SoS should remain constant regardless of the contrast levels. These oscillations and distortions reflect FWI’s sensitivity to complex SoS distributions and its reliance on a solid initial guess. Furthermore, FWI is highly time-consuming, requiring 3200 s to generate a single image, making it impractical for large-scale or real-time applications.

Several aspects of the experimental design are critical for interpreting the FWI performance in this study. The simulations are intentionally constructed to target high-contrast imaging scenarios relevant to both discretized SoS prediction and tissue-level segmentation, where conventional inversion methods are known to exhibit pronounced nonlinearity and convergence challenges [1,31]. In coarsely quantized SoS experiments, the coexistence of low-SoS ($900\;m\cdot s^{-1}$) and high-SoS regions ($2250\;m\cdot s^{-1}$) introduces strong wavefield distortions and increases susceptibility to cycle skipping. In the tissue segmentation setting derived from brain phantoms, even higher contrasts are present, particularly in the cortical skull regions with SoS values in the range of 2800–$2900\;m\cdot s^{-1}$, further exacerbating the inversion difficulty.

In addition, the relatively sparse transducer configuration with 32 elements limits angular diversity and wavefield coverage, thereby compounding the ill-posedness of the inversion problem [31]. This ill-posedness makes FWI particularly sensitive to the choice of the initial guess [1,24]. To ensure consistency across experiments and to isolate architectural effects, the primary FWI baseline is initialized from a homogeneous background. An informed-initialization setting is further investigated, in which the full skull structure, including both cortical and cancellous components, is provided as prior information. Despite this favorable initialization, FWI still struggles to accurately delineate tissue boundaries, highlighting the inherent difficulty of resolving sharp interfaces under high-contrast and reduced-sensor conditions. Collectively, these experimental constraints define a stringent evaluation setting that reflects practical limitations and underscores the robustness of the proposed direct signal-to-segmentation framework in challenging scenarios.

State-of-the-art neural networks for UCT imaging often rely on preprocessing techniques to transform raw sensor data into intermediate representations, such as Time-of-Flight (ToF) measurements or wave propagation models [19,20,21,22]. Although these methods can simplify downstream processing, they introduce assumptions and approximations that may lead to the loss of critical information, such as subtle signal variations in amplitudes or phase shifts. Furthermore, many existing methods have been validated on datasets with a relatively narrow range of SoS values, unlike the broader range of 900 to $2250\;m\cdot s^{-1}$ considered in this study. This raises concerns about their generalizability to more diverse and complex UCT scenarios, particularly those involving materials with extreme SoS values, such as bone (2000 to $4000\;m\cdot s^{-1}$). In comparison, FWI demonstrated consistent performance in low-contrast regions, effectively capturing the basic structures. However, its limitations in high-contrast regions and tendency to produce oscillatory predictions highlight the challenges of extending these approaches to datasets with more significant variability and complexity.

DeepUCT [24], a direct learning-based framework that maps raw ultrasound sensor data to UCT images without preprocessing, has demonstrated strong performance in prior studies. In comparison, the proposed method achieves higher accuracy for both clean and noisy data across discretized SoS prediction and tissue-based segmentation tasks within the evaluated experimental settings. Designed explicitly for UCT reconstruction tasks, the model features a modular multi-input network architecture that aligns with the UCT system by matching the number of input networks with the number of sensors. This modular design supports adaptation to different configurations of UCT machines. When the number of sensors changes, the number of input streams is adjusted accordingly, and the shared input sub-network is applied independently to each stream. If the input dimension changes due to variations in transducer layout or sampling density, only the input layer of the shared sub-network requires modification, while the rest of the network remains unchanged. These characteristics reflect a structurally flexible and scalable architecture that aligns naturally with the distributed and multi-sensor nature of UCT systems.

The proposed CUCT-Net model introduces a multi-input network setup designed to generate distinct perspectives from all receivers to address the two main types of errors, geometrical and classification, as highlighted earlier. By adopting multiple perspectives, this design provides a broader and more detailed understanding of the ROI than DeepUCT. Unlike DeepUCT, which uses a single CNN to process data from 32 receivers as 32 filters, the proposed method treats each receiver’s data separately, thereby enabling a more nuanced analysis. This approach significantly reduces the likelihood of severe classification errors, such as complete negligence of certain regions. In misclassification cases, the multi-input design mitigates errors by incorporating perspectives from all the receivers surrounding the ROI.

Additionally, the model integrates SUs to refine the predictions and enhance geometrical accuracy. SUs employ convolution kernels of varying sizes to extract diverse features from ultrasound signals, thereby enabling more precise representations of the ROI. This combination of detailed feature extraction and multi-perspective design directly addresses geometrical errors, resulting in UCT images with more refined details and improved overall SoS estimation quality.

In Phase 1, CUCT-Net consistently outperforms both FWI and DeepUCT across the Original, Distorted, and Mixed datasets. The Original dataset challenges models with small inner regions and low SoS contrast. By independently processing the signals from all 32 transducers, the CUCT-Net effectively aggregates complementary spatial information, reducing misclassifications in low-contrast regions. The Distorted dataset introduces irregular geometries that require accurate boundary and shape modeling. Although FWI performs adequately in low-contrast regions, it struggles with high-contrast and distorted structures. DeepUCT improves FWI but remains limited in resolving complex contours and closely spaced regions. The incorporation of SUs enables the CUCT-Net to capture multi-scale features more effectively, resulting in improved segmentation of distorted shapes and subtle SoS variations. The Mixed dataset combines both regular and irregular structures, posing a stringent generalization test. In this setting, FWI fails to recover complex structures, and DeepUCT exhibits inconsistent predictions across the expanded SoS range. On the other hand, CUCT-Net maintains robust performance and accurately separates both regular and irregular contours, demonstrating superior generalization across heterogeneous phantom types.

Phase 2 evaluates the robustness of the model under noisy acquisition conditions. FWI experiences significant degradation in both geometric accuracy and class discrimination, particularly in high-contrast regions, highlighting its sensitivity to the noise. DeepUCT exhibits greater robustness but remains constrained by architectural limitations when adapting to noisy data. CUCT-Net Large initially exhibits increased sensitivity to noise owing to its emphasis on fine-grained feature extraction. However, with transfer learning using a small noisy dataset and few training epochs, the model rapidly adapts and recovers a performance comparable to that under clean conditions. This behavior reflects a trade-off between the precision and noise sensitivity. The slight improvement in the SSIM observed for the Distorted dataset under noise is attributed to the larger irregular structures and mild noise-induced smoothing effects, which reduce the relative impact of boundary errors on the structural similarity metrics.

In the Phase 3 Square experiment, CUCT-Net Large maintains high segmentation accuracy despite the change in transducer geometry, demonstrating robustness to different sensor configurations without requiring architectural modification. For the DBB experiment, FWI initialized with a uniform SoS model struggles to resolve tissue boundaries, particularly at high-contrast interfaces, such as the skull and CSF, where reconstructed values are underestimated and misclassified after closest mapping. Providing an informed initial guess (IG) with skull structures (FWI (IG)) improves the overall delineation of the brain region but fails to fully resolve the boundaries between adjacent tissues, especially between the gray and white matter and within thin fat layers. DeepUCT produces visually coherent predictions with a well-preserved global structure, particularly for the outer tissue layers. However, a comparison with the ground truth reveals a tendency to overpredict the white matter at the expense of the gray matter, leading to blurred internal boundaries and reduced Dice and IoU scores, an effect that is subtle in visualization but reflected in the quantitative metrics. CUCT-Net Large more closely matches the ground-truth tissue distribution by reducing white matter overprediction and improving gray–white matter separation while maintaining accurate localization of the outer tissue layers. Nonetheless, minor smoothing and occasional misclassification persist at complex or thin tissue interfaces, reflecting resolution limitations and the intrinsic difficulty of directly segmenting raw sensor data. Overall, these results support the feasibility of direct tissue segmentation with improved robustness under high-contrast and structurally complex conditions. Further improvements are expected with increased training data and higher spatial resolution, which would enable more precise delineation of fine tissue boundaries and closer agreement with the ground truth.

To further analyze CUCT-Net Large’s behavior, representative failure cases corresponding to the lowest mIoU samples are examined. These cases are primarily associated with small brain slices near the superior region, where the anatomical area is reduced. In such scenarios, the segmentation task becomes more challenging due to limited spatial context and increased class imbalance, particularly for thin tissue layers. Quantitatively, the lowest mIoU values range from approximately 0.55 to 0.60, with corresponding Dice scores between 0.69 and 0.73. Despite these lower region-based scores, boundary accuracy remains stable, with Boundary F1 scores above 0.88 and HD95 values within 1.75–1.88. This behavior arises from the fundamental differences between region-based and boundary-based metrics. In small slices, the segmented region occupies a limited number of pixels, making Dice and IoU highly sensitive to small absolute errors, which leads to a noticeable drop in overlap-based scores. In contrast, boundary-based metrics evaluate geometric consistency along object contours and are less sensitive to region size. As long as the predicted boundaries remain close to the ground truth, these metrics remain stable. This indicates that the observed errors are localized and do not compromise the overall structural integrity of the segmentation.

Across the three experimental phases, the CUCT-Net demonstrates consistent behavior under progressively challenging conditions. Phase 1 establishes the model’s effectiveness for quantized high-contrast SoS prediction under clean conditions, while Phase 2 shows that the same formulation can be adapted to noisy measurements through transfer learning with limited additional data. Phase 3 extends this analysis to generalization scenarios, where the Square experiment confirms robustness to changes in transducer geometry, and the DBB experiment demonstrates the feasibility of direct tissue segmentation from raw ultrasound sensor data. Together, these results indicate that the proposed multi-input, segmentation-driven framework can accommodate variations in the acquisition configuration, signal quality, and output definition without fundamental changes to the model architecture. This behavior can be interpreted from both a physical and learning perspective. In UCT, the inverse problem becomes increasingly ill-posed under high contrast, sparse sensing, and complex wave interactions, where small perturbations in measurements can lead to large variations in reconstructed SoS. By reformulating the problem as a direct signal-to-segmentation task with quantized outputs, the proposed method effectively reduces the solution space and avoids the instability associated with continuous inversion. The multi-encoder architecture further supports this formulation by decomposing the measurement space into sensor-aligned representations, allowing the network to capture complementary wavefield information from different acquisition perspectives. Together, these design choices explain the observed robustness and improved performance, particularly in high-contrast and noisy scenarios where conventional inversion methods struggle.

From a broader perspective, these findings suggest that framing UCT as a direct signal-to-segmentation problem provides a viable alternative to conventional inversion-based approaches in high-contrast and sparse sensing regimes. Although the proposed method does not eliminate the challenges associated with complex tissue interfaces or limited resolution, it offers improved robustness compared to FWI and existing learning-based methods, such as DeepUCT, under the evaluated conditions. These results indicate both the strengths and current limitations of the proposed formulation and motivate further investigation of direct segmentation approaches under more complex acquisition settings in the future.

## 5. Limitations and Future Directions

Although the proposed model demonstrates robust performance across different experimental phases, a limitation remains in the use of fixed output classes defined for each task. In the current study, the number of prediction classes is specified according to the target application, such as quantized SoS levels for predefined tissue categories. Although this choice enables stable and interpretable segmentation, it constrains the output resolution to the selected class definitions. Nevertheless, the framework is flexible and can be readily adapted by modifying the output class configuration. For quantized SoS prediction, finer quantization can potentially be achieved by increasing the number of output classes to represent smaller step sizes. This capability is partially validated in the DBB experiments, where tissue-specific SoS values are sampled from continuous ranges and subsequently quantized into tissue classes, resulting in effective SoS differences on the order of only a few to tens of meters per second between neighboring classes. This indicates that the proposed framework is not limited to coarse quantization and can be extended toward finer SoS representations by increasing the class resolution. Similarly, for tissue-level segmentation, the output classes can be adjusted to reflect the number and types of tissues within the region of interest for various anatomical targets. This flexibility provides a clear pathway for extending the proposed approach to broader UCT applications in the future.

The phantoms used in Phases 1 and 2 are intentionally simplified to ensure computational tractability and to support the controlled evaluation of the proposed architecture within a simulation-based framework, whereas the DBB phantoms in Phase 3 exhibit substantially higher structural complexity and irregular tissue geometry. For the DBB experiments, the primary limitation is the relatively low spatial resolution (153×153), which constrains the precise delineation of thin or closely adjacent tissue boundaries. As a transitional step toward more realistic clinical imaging, this study models only a single acoustic parameter (SoS), while additional effects such as attenuation and density are omitted to reduce simulation and training complexity, consistent with prior UCT studies at comparable frequencies [52,53]. Future work will extend the proposed framework to higher-resolution and anatomically realistic phantoms, incorporate multiparameter prediction (e.g., attenuation), and evaluate the performance on real-world datasets as they become available.

The Phase 3 experiment focuses on brain tissue segmentation based on SoS information derived from ultrasound measurements. In this context, the ability to represent pathological conditions is inherently dependent on whether such conditions introduce sufficiently distinct acoustic contrast. Pathologies associated with high contrast, such as hemorrhagic regions [54,55,56], are expected to be more readily detectable, whereas conditions characterized by subtle or smoothly varying SoS changes, such as ischemic stroke or edema, may be more difficult to distinguish due to overlap with normal tissue distributions. This limitation is consistent with findings in quantitative transmission ultrasound imaging, where sound-speed maps provide strong structural contrast but may not fully distinguish complex pathological tissue characteristics without additional parameters [57,58]. Furthermore, the current study is limited to commonly observed anatomical tissues due to the scarcity of publicly available UCT datasets with annotated pathological cases. Extending the framework to include additional classes representing pathological tissues is feasible in principle, but would require carefully curated datasets and training strategies to address challenges such as class imbalance, overlapping acoustic properties, and variability in lesion size, shape, and location. In addition, incorporating complementary acoustic parameters, such as attenuation, may further improve sensitivity to subtle pathological variations, as commonly adopted in quantitative ultrasound imaging systems.

The use of 32 transducers in this study, while effectively demonstrating model adaptability, reflects the resource constraints during simulation and training. Each sample generates a data tensor exceeding one million values, and increasing the number of transducers substantially increases the memory requirements and computational costs. Optimizing the imaging performance under a limited number of transducers is also of practical relevance, as reducing hardware complexity and scan times is often critical for real-world UCT deployments. Achieving high-quality SoS estimation and tissue segmentation using fewer receivers could facilitate the development of more accessible and cost-effective clinical systems. Clinical UCT systems typically employ denser and often irregular transducer arrangements, which can provide richer measurement information and potentially enhance the reconstruction fidelity. Extending the framework to accommodate larger and more complex transducer configurations remains an important direction for future research to validate its robustness and scalability.

In this study, the sources were modeled as point sources that emitted isotropic pressure fields. This simplification was adopted to enable a controlled evaluation of the proposed network architecture under consistent simulation conditions, isolating the effect of network design from additional variability in source characteristics. This approach also aligns with prior UCT studies that employed similar modeling assumptions [9,51]. Future extensions will aim to incorporate more realistic source characteristics, including finite aperture effects and beam directivity, to better reflect the practical behavior of clinical UCT systems.

By addressing these limitations, future studies will enhance the applicability of the model to real-world UCT applications. Planned advancements in phantom complexity, model scalability, and incorporation of real-world data will strengthen the role of deep learning in UCT and further validate the robustness of CUCT-Net for complex imaging scenarios.

## 6. Conclusions

This study presents CUCT-Net, a CNN that formulates UCT as a direct signal-to-image learning problem under two complementary settings: quantized high-contrast SoS estimation and direct tissue-level segmentation. The proposed multi-input architecture fuses features from parallel per-transducer encoders to generate a quantized SoS map, from which a segmented image (tissue classes in the brain experiments) is derived. The integration of SUs enhances multi-scale feature extraction and facilitates accurate mapping from raw sensor data to image-space representations. The performance of CUCT-Net is evaluated through a series of controlled experiments and comparisons with FWI and DeepUCT, demonstrating accurate quantized SoS recovery in high-contrast regimes, robust tissue segmentation in anatomically complex phantoms, strong resilience to noise through transfer learning, and adaptability to varying sensor configurations. Lightweight variants further illustrate the trade-off between model complexity and computational efficiency, achieving sub-second inference times on GPU hardware. Overall, the results demonstrate the feasibility of bypassing iterative inversion by directly inferring quantized acoustic or tissue representations from raw UCT measurements, particularly in reduced-sensor and high-contrast scenarios where conventional FWI can be unstable. Although the present study focuses on quantized outputs, the proposed framework provides a flexible foundation for future extensions to finer SoS quantization and more detailed tissue segmentation; these directions are not evaluated in the current work but motivate continued development of data-driven end-to-end UCT imaging.

## Figures and Tables

**Table 1:** Data split for the three experimental phases. Phase 1 used clean disc-based synthetic phantoms. Phase 2 used noisy disc phantoms for transfer learning. Phase 3 included two setups: a square sensor configuration applied to disc phantoms and circle sensor configuration on DBB phantoms for brain tissue segmentation.

Dataset	Train	Val	Test
Phase 1 (Clean, Disc)	18,000	2000	2000
Phase 2 (Noisy, Disc)	500	250	250
Phase 3 (Square, Disc)	8000	1000	1000
Phase 3 (DBB)	18,000	2000	2000

**Table 2:** Comparison of Size & Parameters.

$\frac{\mathrm{Properties}}{\mathrm{Model}}$	Weight Size	Parameters	GFLOPs	Time
FWI	–	–	–	3200 s
DeepUCT	470 MB	41,072,872	378	0.05 s
CUCT Small	134 MB	11,648,200	340	0.23 s
CUCT Base	177 MB	15,360,264	548	0.28 s
CUCT Large	245 MB	21,292,904	1348	0.73 s

**Table 3:** Quantitative Test Performance of Different Architectures for Original Dataset.

$\frac{\mathrm{Metrics}}{\mathrm{Structures}}$	Dice	Accuracy	IoU	SSIM
FWI	0.5540	0.5320	0.2407	0.3246
DeepUCT	0.9444	0.9500	0.8611	0.9249
CUCT-Net Small	0.9664	0.9677	0.9086	0.9439
CUCT-Net Base	0.9791	0.9802	0.9418	0.9622
CUCT-Net Large	**0.9811**	**0.9819**	**0.9456**	**0.9706**

**Table 4:** Quantitative Test Performance of Different Architectures for Distorted Dataset.

$\frac{\mathrm{Metrics}}{\mathrm{Structures}}$	Dice	Accuracy	IoU	SSIM
FWI	0.6793	0.6525	0.2755	0.4224
DeepUCT	0.9498	0.9568	0.8446	0.9258
CUCT Small	0.9750	0.9775	0.9169	0.9472
CUCT Base	0.9819	0.9833	0.9377	0.9567
CUCT Large	**0.9857**	**0.9867**	**0.9473**	**0.9654**

**Table 5:** Quantitative Test Performance of Different Architectures for Mixed Dataset.

$\frac{\mathrm{Metrics}}{\mathrm{Structures}}$	Dice	Accuracy	IoU	SSIM
FWI	0.6197	0.5623	0.2609	0.4101
DeepUCT	0.9484	0.9555	0.8621	0.9255
CUCT Small	0.9727	0.9757	0.9254	0.9458
CUCT Base	0.9793	0.9813	0.9421	0.9546
CUCT Large	**0.9841**	**0.9857**	**0.9528**	**0.9710**

**Table 6:** Quantitative Test Performances for Noisy Sensor Data.

Model	Dataset	Original				Distorted				Mixed			
	SNR	Dice	Acc	IoU	SSIM	Dice	Acc	IoU	SSIM	Dice	Acc	IoU	SSIM
FWI	20	0.6115	0.5601	0.2365	0.2840	0.6558	0.6373	0.2439	0.2987	0.5828	0.5550	0.2243	0.3453
	30	0.5257	0.5141	0.2241	0.2950	0.6798	0.6754	0.2711	0.3581	0.6121	0.5584	0.2435	0.3882
	40	0.5428	0.4829	0.2144	0.3147	0.6786	0.6681	0.2584	0.4158	0.6133	0.5814	0.1914	0.3639
DeepUCT	20	0.8996	0.9158	0.7742	0.9061	0.9516	0.9591	0.8518	0.9269	0.9323	0.9407	0.8183	0.9153
	30	0.9274	0.9369	0.8278	0.9178	0.9529	0.9600	0.8554	0.9275	0.9376	0.9450	0.8307	0.9181
	40	0.9291	0.9379	0.8304	0.9179	0.9529	0.9602	0.8557	0.9276	0.9378	0.9452	0.8314	0.9187
CUCT Large	20	**0.9738**	**0.9760**	**0.9346**	**0.9497**	**0.9792**	**0.9821**	**0.9333**	**0.9636**	**0.9668**	**0.9713**	**0.9128**	**0.9471**
	30	**0.9783**	**0.9783**	**0.9434**	**0.9600**	**0.9811**	**0.9838**	**0.9391**	**0.9646**	**0.9744**	**0.9779**	**0.9298**	**0.9569**
	40	**0.9803**	**0.9812**	**0.9438**	**0.9678**	**0.9835**	**0.9857**	**0.9447**	**0.9676**	**0.9753**	**0.9780**	**0.9303**	**0.9581**

**Table 7:** Quantitative Test Performance of CUCT-Net Large for Phase 3, Square Dataset.

$\frac{\mathrm{Metrics}}{\mathrm{Structures}}$	Dice	Accuracy	IoU	SSIM
Square	0.9804	0.9816	0.9262	0.9548

**Table 8:** Quantitative Test Performance of Different Architectures for Phase 3, DBB Dataset.

$\frac{\mathrm{Metrics}}{\mathrm{Structures}}$	Dice	Accuracy	IoU	SSIM	HD95	Boundary F1
FWI	0.4126	0.8142	0.3277	0.6952	17.3564	0.3948
FWI (IG)	0.4901	0.8535	0.3951	0.7079	12.5440	0.4691
DeepUCT	0.7160	0.8990	0.5815	0.8257	2.1677	0.8595
CUCT Large	**0.8190**	**0.9224**	**0.7104**	**0.8651**	**1.6831**	**0.9074**

## Data Availability

The code developed and used in this study is openly available on GitHub at https://github.com/K46gqh/CUCT-Net (accessed on 27 April 2026). The dataset analyzed during the current study is not publicly available due to ongoing restrictions, but can be obtained from the corresponding author upon reasonable request at mka9@psu.edu.

## Abbreviations

The following abbreviations are used in this manuscript:

FWI	Full Waveform Inversion
UCT	Ultrasound Computed Tomography
SoS	Speed of Sound
ToF	Time-of-Flight
IR	Iterative Reconstruction
CNN	Convolutional Neural Network
ROI	Region of Interest
PML	Perfectly Matched Layers
